# Observation of the Anomalous Hall Effect in a Layered Polar Semiconductor

**DOI:** 10.1002/advs.202307306

**Published:** 2023-12-08

**Authors:** Seo‐Jin Kim, Jihang Zhu, Mario M. Piva, Marcus Schmidt, Dorsa Fartab, Andrew P. Mackenzie, Michael Baenitz, Michael Nicklas, Helge Rosner, Ashley M. Cook, Rafael González‐Hernández, Libor Šmejkal, Haijing Zhang

**Affiliations:** ^1^ Max Planck Institute for Chemical Physics of Solids 01187 Dresden Germany; ^2^ Max Planck Institute for the Physics of Complex Systems 01187 Dresden Germany; ^3^ Scottish Universities Physics Alliance School of Physics and Astronomy University of St Andrews St Andrews KY16 9SS United Kingdom; ^4^ Institut für Physik Johannes Gutenberg Universität Mainz 55128 Mainz Germany; ^5^ Grupo de Investigación en Física Aplicada Departamento de Física Universidad del Norte Barranquilla 080020 Colombia; ^6^ Institute of Physics Czech Academy of Sciences Cukrovarnická 10 Praha 6 162 00 Czech Republic

**Keywords:** anomalous Hall effect, Berry curvature, ionic gating, magnetism, polar structure

## Abstract

Progress in magnetoelectric materials is hindered by apparently contradictory requirements for time‐reversal symmetry broken and polar ferroelectric electronic structure in common ferromagnets and antiferromagnets. Alternative routes can be provided by recent discoveries of a time‐reversal symmetry breaking anomalous Hall effect (AHE) in noncollinear magnets and altermagnets, but hitherto reported bulk materials are not polar. Here, the authors report the observation of a spontaneous AHE in doped AgCrSe_2_, a layered polar semiconductor with an antiferromagnetic coupling between Cr spins in adjacent layers. The anomalous Hall resistivity 3 μΩcm is comparable to the largest observed in compensated magnetic systems to date, and is rapidly switched off when the angle of an applied magnetic field is rotated to ≈80° from the crystalline *c*‐axis. The ionic gating experiments show that the anomalous Hall conductivity magnitude can be enhanced by modulating the *p*‐type carrier density. They also present theoretical results that suggest the AHE is driven by Berry curvature due to noncollinear antiferromagnetic correlations among Cr spins, which are consistent with the previously suggested magnetic ordering in AgCrSe_2_. The results open the possibility to study the interplay of magnetic and ferroelectric‐like responses in this fascinating class of materials.

## Graphical abstract

A spontaneous anomalous Hall effect is observed in a layered polar semiconductor, and, remarkably, the magnitude of the anomalous Hall conductivity can be enhanced by tuning the carrier density employing ionic‐gated transistors. Theoretical calculations, considering the magnetic order and polar nature, align well with experimental observations. The findings shed new light on exploring the interplay between magnetic and polar structures.

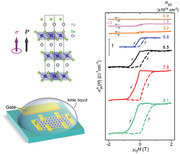

## 1 Introduction

The anomalous Hall effect (AHE), in which electrons acquire a transverse velocity relative to an applied electric field in the absence of a magnetic field, is one of the most fundamental phenomena in condensed matter physics.^[^ ^1^, ^2^ ^]^ Developments in theory based on Berry‐phase concepts have provided a comprehensive framework for understanding the AHE, which may occur not only in ferromagnets, but also more generally in magnetically compensated systems with broken time‐reversal symmetry (TRS) in their momentum space electronic structure, such as noncollinear kagome magnets and altermagnets.^[^ ^3^, ^4^, ^5^, ^6^, ^7^, ^8^, ^9^, ^10^, ^11^ ^]^

Among many predictions and observations in this rapidly‐moving field, the case of an AHE associated with a polar structure stands out. Polar materials can exhibit ferroelectricity and spin‐orbit interaction induced spin polarization.^[^ ^12^, ^13^ ^]^ When TRS breaking in electronic structure coexists in such a system, the interplay between magnetic order and polarity creates a promising platform for the development of spintronic and magnetoelectric devices with rich functionality.^[^ ^14^, ^15^, ^16^ ^]^ Although there are reports that combine magnetic order and polarity at the interfaces or in heterostructures,^[^ ^14^, ^17^ ^]^ it is a formidable task to realize the coexistence in a single bulk material platform.

In terms of material physics, a minimal ingredient for such an observation is a system with a crystal and magnetic structure that allows for the coexistence of a polar vector, ***P***, and a Hall pseudovector, $\sigma =(\sigma_{yz},\sigma_{zx},\sigma_{xy})$, where the components represent anomalous Hall conductivities.^[^ ^2^, ^18^, ^19^ ^]^ Particularly interesting classes with polar interfaces intrinsic to their layered structure include layered triangular‐lattice delafossite systems.^[^ ^20^ ^]^ Previous work has reported ferroelectricity in AgCrS_2_ and CuCrS_2_—layered magnetic semiconductors that have a similar triangular‐lattice framework.^[^ ^21^, ^22^, ^23^ ^]^ This suggests that a large effect may be visible in related layered polar materials, and an outstanding question arises whether this class of crystals can also exhibit an AHE.

Here, we report that one such a doped semiconductor, AgCrSe_2_, fulfills the symmetry requirements of polar crystal structure, and shows a spontaneous AHE. We demonstrate that it is an intrinsic AHE by comparing its evolution to that of the magnetization, and perform a consistency check by showing that the magnitude of the anomalous Hall conductivity is temperature‐ and scattering‐independent below 50 K. We also report and discuss the observation of a rather pronounced plateau in the Hall resistivity as a function of the angle of the applied field relative to the crystalline *c*‐axis, and show that the magnitude of the observed AHE is tunable by the application of an ionic gate.

## 2 Results

**Figure 1:**
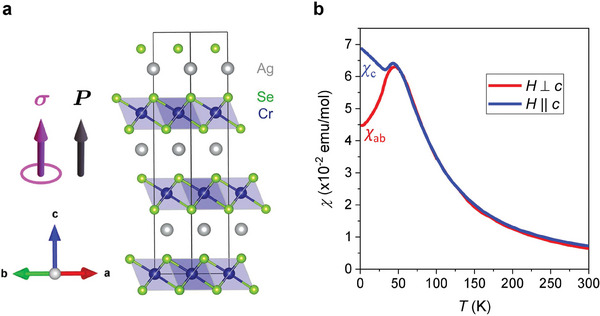
Crystal structure and magnetic susceptibility of AgCrSe_2_. a) Crystal structure of AgCrSe_2_ (space group *R3m*). The polarization direction ***P*** and the symmetry allowed Hall pseudovector ***σ*** (corresponding to an anomalous Hall conductivity σ_*xy*_) are marked. b) Magnetic susceptibility measured with an applied 1 T magnetic field along and perpendicular to the *c*‐axis, respectively.

AgCrSe_2_ has a layered structure with alternate Ag layers and edge‐sharing CrSe_6_ octahedral layers repeating along the *c*‐axis,^[^ ^24^, ^25^, ^26^, ^27^, ^28^ ^]^ as illustrated in **Figure** **1**a. The compound crystallizes into the noncentrosymmetric *R3m* space group. The polar structure is realized by the alternating layers of Ag and CrSe_6_, which break the inversion symmetry and allow for a polarization direction along the *c*‐axis (Figure 1a).^[^ ^21^, ^22^, ^23^, ^29^ ^]^ The Cr atoms in each layer form a triangular lattice and host *S* = 3/2 spins. Previous neutron diffraction characterization and magnetization measurements revealed that the adjacent octahedral layers couple antiferromagnetically. A noncollinear spin structure in the *ab*‐plane has been reported,^[^ ^27^, ^30^, ^31^ ^]^ revealing interplay between various channels of intralayer exchange couplings. In Figure 1b we present the temperature dependence of the magnetic susceptibility χ of a AgCrSe_2_ single crystal, measured with a magnetic field applied along the *c*‐axis (χ_*c*_) and in the *ab*‐plane (χ_*ab*_), respectively. The susceptibility is isotropic at high temperatures, but χ_*ab*_ and χ_*c*_ deviate from each other below T* = 50 K, the characteristic temperature at which spin order begins to become long‐ranged.

For the magneto‐transport measurements, we realized microfabricated devices based on exfoliated AgCrSe_2_ crystals with thicknesses ranging from 100 to 800 nm and compared our results with those from bulk single crystal devices. As expected in a material with a layered crystal structure, the resistive anisotropy $\rho_{zz}^{TOT}/\rho_{xx}^{TOT}$ is large, rising from 25 at 50 K to 100 at 2 K, and the in‐plane resistivity at 2 K is approximately 2 mΩcm (see Section SII, Supporting Information), which may be the result of doping due to an intrinsic non‐stoichiometry. Throughout the paper, we map the Cartesian coordinates commonly used to describe the Hall effect to the crystalline ones, with the *z* axis being the crystalline *c* axis, the *xy* plane being the *ab* plane of the crystalline layers. The large resistive anisotropy indicates a nearly 2D electronic structure, justifying the use of 2D expressions in the discussion.

**Figure 2:**
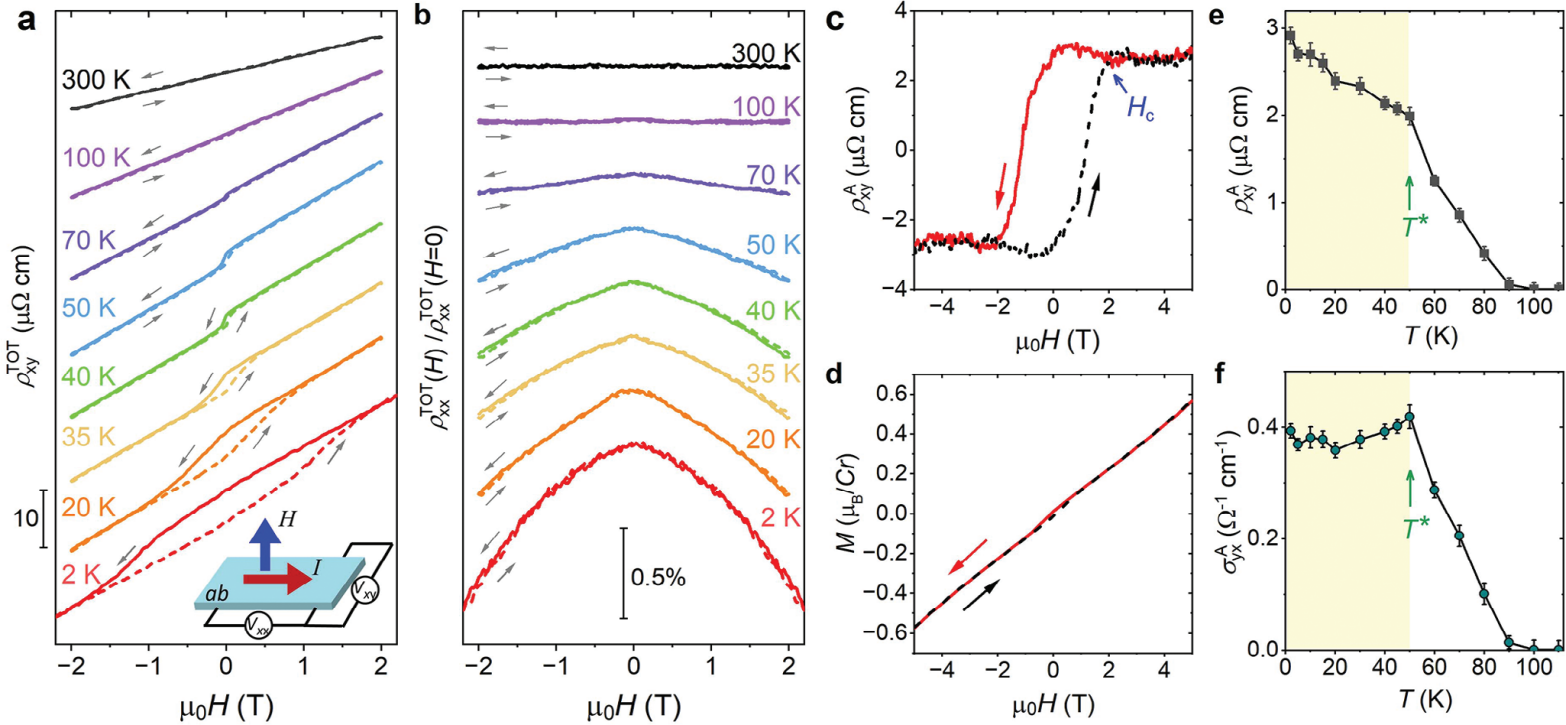
Spontaneous AHE observed in AgCrSe_2_. a) Hall resistivity ($\rho_{xy}^{TOT}\left(H\right)$), as well as b) normalized longitudinal resistivity ($\rho_{xx}^{TOT}\left(H\right)/\rho_{xx}^{TOT}\left(H=0\right)$), measured simultaneously with *H* applied along the *c*‐axis. The curves are offset vertically for clarity. Inset of a): a schematic illustrating the measurement setup. c) $\rho_{xy}^{A}=\rho_{xy}^{TOT}-\rho_{xy}^{0}$ is plotted as a function of *H* at 2 K. The resistivity shows a clear jump and saturates at a critical field *H*_*c*_. d) Magnetization *M* versus*H* measured at 2 K with the magnetic field applied along the *c*‐axis. e) Temperature evolution of the zero‐field component $\rho_{xy}^{A}\left(H=0\right)$ and f) the corresponding $\sigma_{yx}^{A}$ obtained by inverting the resistivity tensor. Error bars include the uncertainty in extracting the zero‐field resistivity from the Hall measurements.

We first discuss our main experimental evidence for the AHE, which was observed by measuring the Hall resistivity $\rho_{xy}^{TOT}$ upon applying the magnetic field *H* perpendicular to the *ab*‐plane. The measurements were carried out employing the setup, as illustrated by the schematic in the inset of **Figure** **2**a. From 300 to 100 K, the Hall resistivity $\rho_{xy}^{TOT}$ is linear as a function of *H* (Figure 2a). The Hall coefficient is positive, indicating that the majority of charge carriers in the system are holes. With further cooling, $\rho_{xy}^{TOT}$ exhibits a clear hysteresis loop with a sizable jump and width 2*H*_*c*_, when the magnetic field is swept back and forth. The Hall resistivity jump and *H*_*c*_ become more pronounced upon lowering the temperature. In contrast to the presence of large jumps in $\rho_{xy}^{TOT}$, the longitudinal resistivity $\rho_{xx}^{TOT}$ measured concomitantly does not exhibit significant variations in this temperature regime, as shown in Figure 2b.

A common question regarding hysteretic AHE signals is their relationship to the magnetization. Because the Hall effect can have a variety of origins, the total Hall conductivity $\sigma_{xy}^{TOT}$ can be expressed as a sum of contributions: $\sigma_{xy}^{TOT}=\sigma_{xy}^{0}+\sigma_{xy}^{A}$, where $\sigma_{xy}^{0}$ is the traditional Hall conductivity from orbital electronic motion and $\sigma_{xy}^{A}$ is the term resulting from *k*‐space Berry curvature. Written in terms of the measured quantities which are resistivities, $\sigma_{yx}^{TOT}=\rho_{xy}^{TOT}/\left({\left(\rho_{xy}^{TOT}\right)}^{2}+{\left(\rho_{xx}^{TOT}\right)}^{2}\right)$. If (and only if) ${\left(\rho_{xx}^{TOT}\right)}^{2}\gg {\left(\rho_{xy}^{TOT}\right)}^{2}$ and $\rho_{xx}^{TOT}$ has a weak magnetic field dependence (see Section SIV, Supporting Information for more details), a similar separation can be made, to a good approximation, in the Hall resistivity:

(1) $$\rho_{xy}^{TOT}\left(H\right)=R_{0}\mu_{0}H+\rho_{xy}^{A}\;$$

here, *R*_0_ is the ordinary Hall coefficient, μ_0_ is the permeability, and we use $\rho_{xy}^{0}$ to represent *R*_0_μ_0_ *H*. In our AgCrSe_2_ microcrystals, $\rho_{xx}^{TOT}$ varies between 1.5 and 3 mΩcm between 2 and 300 K, with a magnetoresistance of less than 2% for μ_0_ *H* < 4 T. Inspection of Figure 2 shows that the above condition is therefore very well satisfied, so the decomposition of Equation (1) is justified.

The field‐linear part of $\rho_{xy}^{TOT}\left(H\right)$ enables the identification of *R*_0_, and the subtraction of $\rho_{xy}^{0}$. As shown in Figure 2c, the anomalous Hall resistivity, $\rho_{xy}^{A}$, is seen to be hysteretic with *H*_*c*_ = 2 T at 2 K and a sizable resistivity jump of ≅6µΩ cm. We also measure the magnetization *M* as a function of the applied field, and plot it in Figure 2d. Clearly, the large hysteresis shown in $\rho_{xy}^{A}$ (Figure 2c) cannot be explained by the linear contribution from *M* (Figure 2d), which is distinct from the conventional ferromagnetism.

One diagnostic sometimes used for the existence of an intrinsic AHE is an anomalous Hall conductivity, $\sigma_{yx}^{A}$, that is independent of scattering.^[^ ^1^ ^]^ In Figure 2e we show the temperature dependence of the zero‐field component $\rho_{xy}^{A}\left(H=0\right)$. The corresponding $\sigma_{yx}^{A}\left(H=0\right)$ obtained by inverting the resistivity tensor $\sigma_{yx}^{A}=\rho_{xy}^{A}/\left({\left(\rho_{xx}^{TOT}\right)}^{2}+{\left(\rho_{xy}^{A}\right)}^{2}\right)$, is plotted in Figure 2f, and $\sigma_{yx}^{A}$ is found to saturate. The fact that $\sigma_{yx}^{A}$ remains constant over a range of temperature in which $\sigma_{xx}^{TOT}$ and hence $\rho_{xy}^{A}$ are both temperature dependent is consistent with the scattering rate independence expected for a momentum‐space Berry‐curvature related effect, but does not rule out one extrinsic mechanism, namely side‐jump scattering.

**Figure 3:**
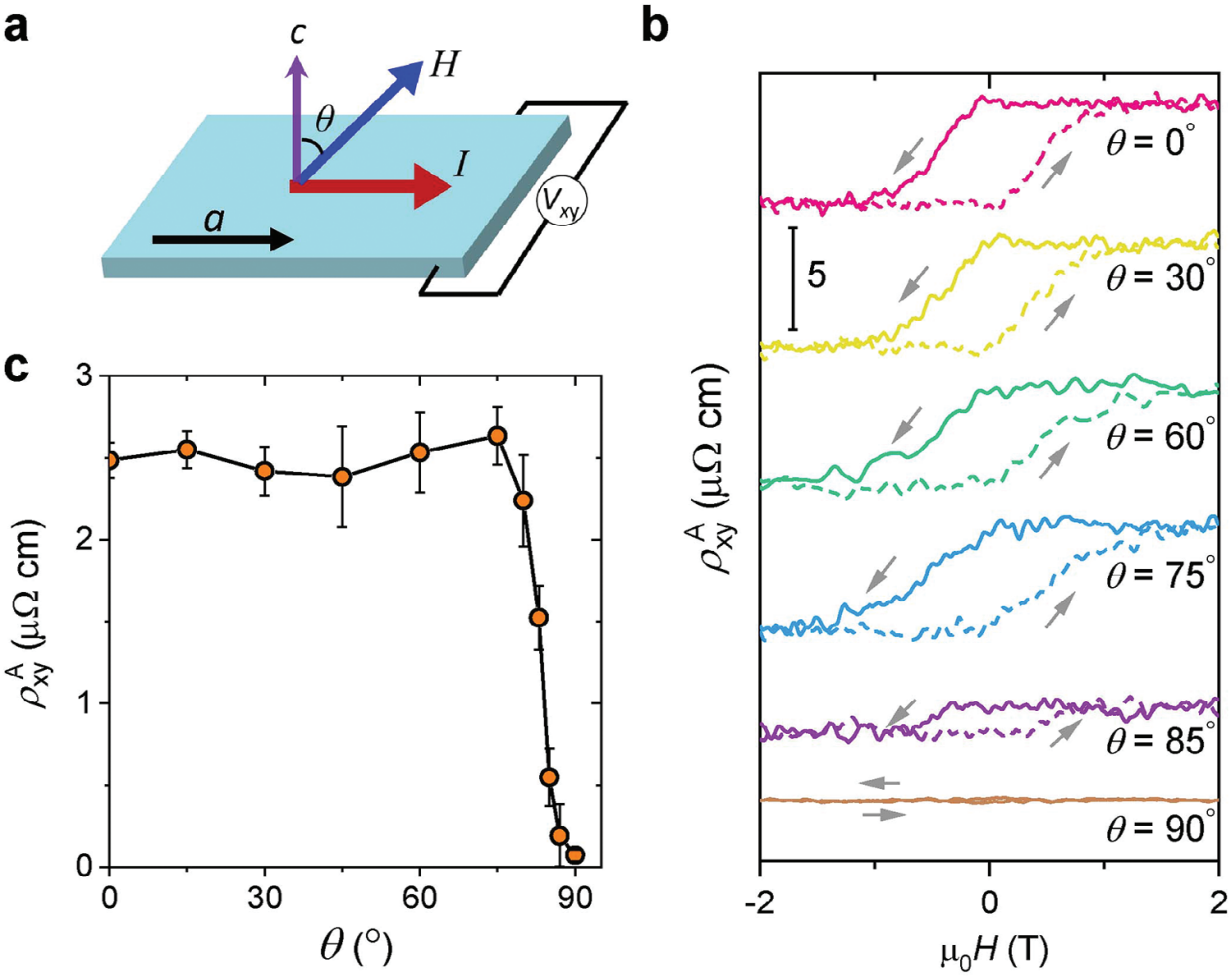
Angular dependence of the AHE. a) Illustration of the measurement setup for the angular dependent AHE. The current is applied in the *ab*‐plane, and the magnetic field is rotating relative to the *c*‐axis. b) $\rho_{xy}^{A}\left(H\right)$ measured at different rotation angles at fixed temperature 5 K. c) Angular dependence of the zero‐field resistivity $\rho_{xy}^{A}$. $\rho_{xy}^{A}$ remains a plateau up to θ ≈ 80°, and then drops abruptly to near zero when *H* is aligned to the *ab*‐plane (θ = 90°). Error bars reflect an estimate of the uncertainty in extracting the resistivity jumps.

The data shown in Figure 2 establish one of our key experimental findings, namely the existence of an AHE in AgCrSe_2_. In order to investigate further, we study the dependence of $\rho_{xy}^{A}$ on the angle of the magnetic field relative to the crystalline *c*‐axis, rotating it by angle θ in the plane of the *c*‐axis and the applied current, as shown in **Figure** **3**a. The result, shown in Figure 3b and summarized in Figure 3c, is striking: $\rho_{xy}^{A}$ remains approximately angle‐independent before ‘switching off’ for θ > 80°. We present this as an empirical fact that will merit further detailed investigation in future, but in the Supporting Information examine and rule out the possibility of a quantized topological origin for it.

**Figure 4:**
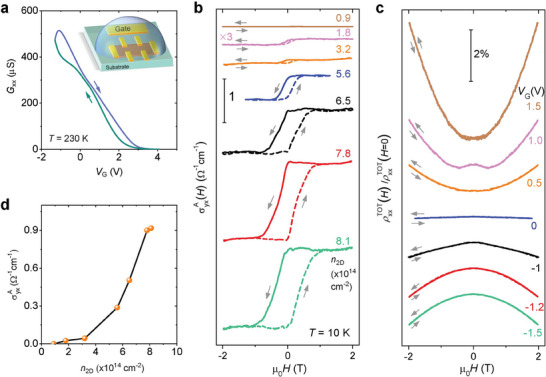
Gate‐tunable AHE in a AgCrSe_2_ thin flake. a) Conductance as a function of the applied gate voltage measured at 230 K. The inset shows a schematic of the device measurement setup. The ionic liquid (DEME‐TFSI) covers both the side gate electrode and the thin flake. b) $\sigma_{yx}^{A}\left(H\right)$ and c) normalized longitudinal resistivity ($\rho_{xx}^{TOT}\left(H\right)/\rho_{xx}^{TOT}\left(H=0\right)$) modulated by the ionic gating. The data were obtained at *T* = 10 K with the magnetic field applied along the *c*‐axis. The applied $V_{G}$ and the carrier density *n*_2*D*_ extracted from the field linear part of the Hall effect at each *V*_*G*_ are labeled beside the curves. d) $\sigma_{yx}^{A}$ as a function of the doping level.

Another fruitful line of investigation is to apply an ionic gate employing ionic gated field effect transistors.^[^ ^32^, ^33^ ^]^ We now demonstrate that an ionic gate drastically modulates the AHE. We employ an ionic field effect transistor setup, in the configuration schematically illustrated in the inset of **Figure** **4**a. The device includes an exfoliated AgCrSe_2_ thin flake, a large‐area side gate pad, as well as the ionic liquid that covers both the thin flake and the gate electrode. Due to the screening effect, the induced conductivity takes place in the surface layers of the material. Figure 4a shows the in‐plane conductance tuned as a function of applied gate voltage, *V*_*G*_. A moderate *V*_*G*_ of a few volts can change the conductance by orders of magnitude. The high doping level of an ionic gate also leads to a drastic change in the AHE, and we show the results of such experiments in Figure 4b,c and d.

In the experiment, we applied both negative and positive gate voltages, producing sheet carrier densities per layer that are both smaller and larger than the carrier density per layer of the bulk devices. As seen in Figure 4b, an AHE with similar characteristics to that of the bulk is observed, with a magnitude that is tuned to be larger (at densities of 6.5, 7.8 and 8.1 × 10^14^ cm^−2^) and smaller than that observed in the bulk. As with the bulk case, the pronounced hysteresis seen in the AHE is absent in the magnetoresistance, shown in Figure 4c. The AHE is modulated to be vanishing at carrier densities smaller than 1.8 × 10^14^ cm^−2^.

Because of screening in a conducting system, the ionic gating investigates a very thin surface layer whose response is very strongly 2D. The qualitative similarity of the responses seen in the gated system to that from the bulk crystal gives further confidence in the 2D nature of the bulk response, which is due to the anisotropy of its electronic structure.

## 3 Discussion

Separating side‐jump scattering contributions from Berry curvature ones to the AHE is a major challenge, as discussed in Ref. [1]. As stated in that review, the pragmatic approach is to investigate whether Berry curvature can account for the observations or not. In the present case, the strong dependence of the AHE magnitude on gating seems intuitively consistent with an energy‐dependent Berry curvature in a band, so we set out to test this with an explicit model.

We start from the symmetry analysis to look for necessary conditions for the occurrence of a nonzero anomalous Hall conductivity, i.e., σ_*xy*_. The out‐of‐plane spin tilting that is favored by Dzyaloshinsky‐Moriya interaction^[^ ^34^, ^35^ ^]^ can, in principle, break symmetries in the crystal and give rise to a ferromagnetic behavior. However, as previously discussed and detailed in the Supporting Information, the resulting net moment alone cannot fully explain the observed large hysteresis loop in the Hall resistivity, and an additional component in the Hall resistivity is still observed after taking the moment into account. Therefore, we resort to other necessary conditions that can possibly explain the observed phenomena.

**Figure 5:**
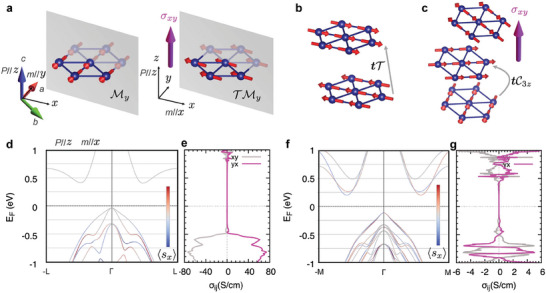
Theoretical calculations of AHE in AgCrSe_2_. a) Model of ferromagnetic Cr atoms in a monolayer. The mirror symmetry plane $M_{y}$ is marked in gray color. The mirror symmetry translation coupled with time reversal symmetry $TM_{y}$ allows for a Hall vector (right panel). b) Model of Cr atoms in the collinear antiferromagnetic states. The unit cell translation coupled with time reversal tT cancels the Hall vector. c) Model of Cr atoms in the noncollinear states. The unit cell translation coupled with rotation symmetry $tC_{3z}$ is marked. d) Spin‐projected energy bands and e) energy dependent anomalous Hall conductivity in ferromagnetic states with spins aligned parallel to the *x*‐axis. f) Spin‐projected energy bands and g) energy dependent anomalous Hall conductivity in antiferromagnetic states in a simplified noncollinear structure. The calculations, for stoichimetric AgCrSe_2_, put the Fermi level in the bandgap. In the real crystals, non‐stoichiometry places it in the hole bands, where the calculations give a finite σ_*xy*_ with a strong doping dependence, qualitatively in accord with the results of our gating experiments.

Inspection of the magnetic space group for various possible magnetic orderings reveals that an anomalous Hall conductivity can be generated either by (a) an in‐plane magnetic moment^[^ ^36^ ^]^ and/or (b) by the noncollinear spin structure.^[^ ^37^ ^]^ As shown in the left panel of **Figure** **5**a, for spins parallel to the *y*‐axis (m//y), the mirror symmetry $M_{y}$ is retained, which excludes the *z*‐Hall vector component. It is worth noting that the Hall conductivity components correspond to the antisymmetric part of the 2nd rank conductivity tensor and thus we can write them as components of a pseudovector, Hall vector, $\sigma =(\sigma_{yz},\sigma_{zx},\sigma_{xy})$.^[^ ^2^, ^18^ ^]^ The cancellation of σ_*xy*_ by $M_{y}$ symmetry operation is consistent with the observed ‘switching off’ of AHE when the magnetic field is aligned along the *a*‐axis, as presented in Figure 3. In contrast, for spins parallel to the *x*‐axis (m//x), the mirror symmetry is augmented by time‐reversal $TM_{y}$, which allows for a Hall vector component σ_*xy*_ that is perpendicular to the *xy*‐plane (right panel of Figure 5a), as well as the component σ_*yz*_. Furthermore, we consider two types of antiferromagnetic states and show that while simple collinear antiferromagnetic ordering cannot explain the observed signal, a nonzero anomalous Hall conductivity is anticipated in the noncollinear antiferromagnetic states for the AgCrSe_2_ crystal structure. We construct the collinear antiferromagnetic state by doubling of the unit cell along the *c*‐axis. In the collinear antiferromagnetic states, the Cr sublattices are connected by the unit cell translation combined with time‐reversal, tT symmetry, as shown in Figure 5b, which forces the Hall vector to vanish. In contrast, the noncollinear antiferromagnetic state (inherent to the experimentally indicated cycloidal spin structure^[^ ^27^ ^]^) can break TRS in electronic structure and lift Kramers spin degeneracy.^[^ ^37^ ^]^ To emulate the effect of noncollinear antiferromagnetic ordering we have considered in our calculations a simplified noncollinear spin structure, as shown in Figure 5c, constructed by tripling the unit cell along the *c*‐axis. The noncollinear antiferromagnetic sublattices are, in this case, related by three fold rotation combined with translation, $tC_{3z}$, and thus allow a Hall vector perpendicular to the *xy*‐plane.^[^ ^2^, ^4^ ^]^

To investigate the interplay between the magnetic order and the polar structure, as well as to corroborate the aforementioned symmetry analysis, we performed density functional theory (DFT) calculations including spin‐orbit coupling and calculated the anomalous Hall conductivity based on the relativistic DFT band structures. The obtained results for various spin configurations are plotted in Figure 5d–g. For spins aligned parallel to the *x*‐axis (right panel of Figure 5a), the energy bands, as shown in Figure 5d, are spin split along the high‐symmetry axes and asymmetric to Γ point. This asymmetric band dispersion is a direct manifestation of the interplay between the polarization and the magnetic order: the polar structure introduces a Rashba‐like spin‐splitting and the magnetic moment along the *x*‐axis (m//x) leads to an asymmetric deformation (a schematic illustration is shown in Section SVIII, Supporting Information). As a result of the band dispersion asymmetry, TRS is broken and an anomalous Hall conductivity up to 80 Scm^‐1^ is obtained for energy levels lower in the valence band (Figure 5e). In addition, for the simplified noncollinear spin structure in the antiferromagnetic states, as shown in Figure 5c, the bands once again exhibit TRS breaking. The calculated band structure shown in Figure 5f highlights the polar, noncentrosymmetric origin of the dominant spin‐orbit coupling, which, in conjunction with the magnetic ordering, gives rise to the AHE.^[^ ^2^ ^]^ We observed a pronounced antisymmetric spin‐polarization (*E*_↑_(**k**) = *E*_↓_(− **k**)), which is distinct from the symmetric spin polarization (*E*_↑(↓)_(**k**) = *E*_↑(↓)_(− **k**)) reported in prior studies of AHEs in centrosymmetric altermagnets^[^ ^4^ ^]^ and kagome magnets.^[^ ^37^ ^]^ The calculations presented in Figure 5g yield the order of magnitude of σ_*xy*_ (≅1 Scm^‐1^) that aligns well with the experimental data, and capture the increase in Hall conductivity as the Fermi level lowers toward the bottom of the valence bands in the range probed by the gating experiments.

The above‐described models are based on coplanar spin configurations that generate a Hall vector perpendicular to the plane. This is distinct from the conventional ferromagnetism that a Hall vector is generated by a moment along the same direction. The scenario involving a more complex noncollinear spin structure has been examined on model level and detailed in Section SIX (Supporting Information), and is consistent with the main conclusions based on DFT results. We emphasize that these models discuss possible origins of the Hall signal only. Nevertheless, our theoretical analysis and calculations demonstrate that the AgCrSe_2_ crystal structure family can host intriguing in‐plane magnetization^[^ ^36^, ^38^, ^39^, ^40^ ^]^ or noncollinear magnetism^[^ ^37^ ^]^ driven AHE. It also demonstrates that the kind of bands that exist in AgCrSe_2_ can yield the anomalous Hall signals with a magnitude consistent with our observations and and gating dependence. We believe that our combined experimental and theoretical study provides good evidence that the AHE in AgCrSe_2_ is both intrinsic and influenced by the polar structure of the material.

## 4 Conclusion

In conclusion, we have observed a spontaneous AHE in the polar, layered, triangular‐lattice material AgCrSe_2_, and have shown how its magnitude can be tuned by an ionic gate. Although the measured anomalous Hall resistivity is comparable with the largest observed in any magnetic material, the anomalous Hall conductivity is far from the quantized value. We show that it can be substantiated by symmetry analysis and DFT calculations. However, the microscopic origin of the TRS breaking remains to be understood, as does the hysteresis in the AHE. Our work motivates further theoretical investigation and detailed neutron scattering studies of this fascinating material class, which we have demonstrated to be an appealing candidate for the coexistence of ferroelectric‐ and ferromagnetic‐like responses and functionalities. Moreover, the gate‐controllable AHE in this material provides a new possibility for the local manipulation of spin states, which can facilitate the realization of stable and compact spintronic and magnetoelectric devices.

## Conflict of Interest

The authors declare no conflict of interest.

## Supporting information

Supporting Information

## Data Availability

The data that support the findings of this study are available from the corresponding author upon reasonable request.
