# Supplementary material for: Observation of the Anomalous Hall Effect in a Layered Polar Semiconductor
Source: Adv Sci (Weinh). 2023 Dec 8;11(6):2307306. doi: 10.1002/advs.202307306 (PMC10853720; doi:10.1002/advs.202307306)
Supplement: Supplementary file 1 — Supporting Information [file ADVS-11-2307306-s001.pdf]

ADVANCED SCIENCE Open Access

Supporting Information

for *Adv. Sci.*, DOI 10.1002/advs.202307306

Observation of the Anomalous Hall Effect in a Layered Polar Semiconductor

Seo-Jin Kim, Jihang Zhu, Mario M. Piva, Marcus Schmidt, Dorsa Fartab, Andrew P. Mackenzie, Michael Baenitz, Michael Nicklas, Helge Rosner, Ashley M. Cook, Rafael González-Hernández, Libor Šmejkal* and Haijing Zhang**

Supporting Information for
Observation of the anomalous Hall effect in a layered polar semiconductor

Seo-Jin Kim,[1] Jihang Zhu,[2] Mario M. Piva,[1] Marcus Schmidt,[1] Dorsa Fartab,[1] Andrew P. Mackenzie,[1,3,*] Michael Baenitz,[1] Michael Nicklas,[1] Helge Rosner,[1] Ashley M. Cook,[1,2] Rafael González-Hernández,[4,5] Libor Šmejkal,[4,6,†] and Haijing Zhang[1,‡]

[1]*Max Planck Institute for Chemical Physics of Solids, 01187 Dresden, Germany*
[2]*Max Planck Institute for the Physics of Complex Systems, 01187 Dresden, Germany*
[3]*Scottish Universities Physics Alliance, School of Physics and Astronomy, University of St Andrews, St Andrews KY16 9SS, United Kingdom*
[4]*Institut für Physik, Johannes Gutenberg Universität Mainz, 55128 Mainz, Germany*
[5]*Grupo de Investigación en Física Aplicada, Departamento de Física, Universidad del Norte, Barranquilla, Colombia*
[6]*Institute of Physics, Czech Academy of Sciences, Cukrovarnická 10, 162 00 Praha 6, Czech Republic*

In this Supporting Information, we describe the device fabrication, details of our measurements and of our theoretical calculations. We also provide additional information that are relevant for the interpretation of our data.

I. MATERIALS AND METHODS

Device preparation

$AgCrSe_2$ single crystals were grown via chemical vapor transport using procedures described in ref. [S1]. The good crystalline quality of as grown crystals was confirmed by X-ray powder diffraction, Laue X-ray diffraction and differential scanning calorimetry.

For the electrical transport measurements, we realized microfabricated devices based on exfoliated crystals (Fig. S1), which were obtained by mechanical exfoliation from bulk crystals and then transferred onto the silicon substrates capped with 280 nm silicon dioxide. Compared with van der Waals materials like graphite, $AgCrSe_2$ is much harder to peel off and the chance of obtaining thin layers is lower. Exfoliated crystals of rectangular shape and uniform thickness were selected and their thicknesses were measured by an atomic force microscope (AFM). Electrical contacts were made by micro-fabrication processes based on electron beam lithography, sputtering of Au, and lift-off. Fig. S1A shows an optical image of a typical device. Fig. S1B shows the corresponding AFM mapping which yields a thickness of ∼430 nm (see Fig. S1C). The gate-tuned devices include an exfoliated $AgCrSe_2$ thin flake, a large-area side gate pad, as well as the ionic liquid that covers both the thin flake and the gate electrode. The gated experiment were only carried out on very thin flakes (typically 40 - 100 nm thick). The ionic liquid that was used for our measurement is N,N-Diethyl-N-methyl-N-(2-methoxyethyl)ammonium bis(trifluoromethanesulfonyl)imide (DEME-TFSI).

To cross-check the results, we also realized devices based on single crystals. A single crystal of length ∼2 mm, width ∼1 mm and thickness ∼50 μm was selected and glued onto a sapphire substrate. Then contacts were deposited by sputtering Au with the crystal covered by a shadow mask, yielding a device similar to the configuration shown in Fig. S1 but on a larger scale.

* andy.mackenzie@cpfs.mpg.de
† lsmejkal@uni-mainz.de
‡ haijing.zhang@cpfs.mpg.de

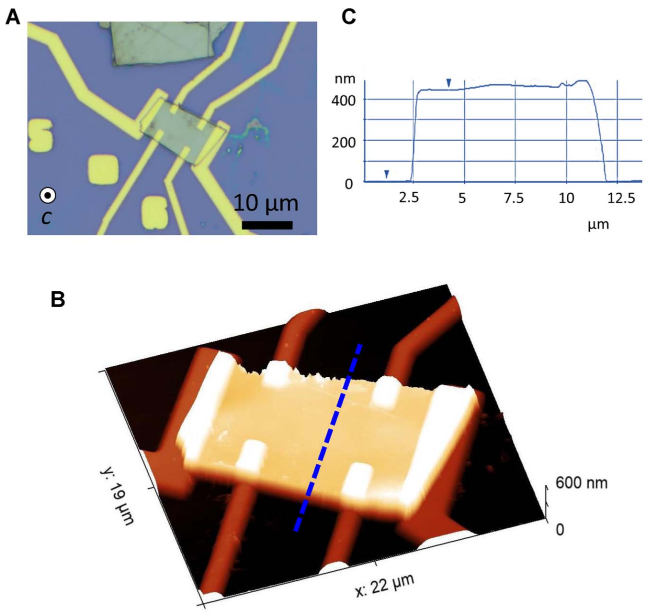

FIG. S1. (A) An optical image of a typical microfabricated device based on an exfoliated crystal. (B) AFM topography of the same device. (C) The height profile of the exfoliated crystal corresponding to the dashed line in (B).

Transport and magnetization measurements

Transport measurements were carried out in a Helium-4 cryostat, equipped with a 9 T magnet. SR830 lock-in amplifiers were used to perform four-point resistance measurements. An alternating current I (100 nA $\sim$ 5 μA) was applied between the source and drain contacts, and the transverse and longitudinal voltages (V_{xy}/V_{xx}) were measured simultaneously. The Hall resistivity was extracted by $\rho_{xy}^{TOT} = tV_{xy}/I$ and the longitudinal resistivity was extracted by $\rho_{xx}^{TOT} = WtV_{xx}/(IL)$, where W is the width, L is the length, and t is the thickness of the device. We used the standard procedure to symmetrize $(\rho_{xx}^{TOT}(H_{\rightarrow}) + \rho_{xx}^{TOT}(-H_{\leftarrow}))/2$ and antisymmetrize $(\rho_{xy}^{TOT}(H_{\rightarrow}) - \rho_{xy}^{TOT}(-H_{\leftarrow}))/2$ the measured ρ_{xx}^{TOT} and ρ_{xy}^{TOT} under positive and negative magnetic fields, and $H_{\rightarrow(\leftarrow)}$ refers to the field sweep direction. Magnetization measurements were performed on as-grown single crystals using a commercial vibrating sample magnetometer (VSM, Quantum Design).

DFT calculations

We have calculated the relativistic DFT band structure of $AgCrSe_2$ in the pseudopotential code Vienna Ab initio Simulation Package (VASP) [S2], within Perdew–Burke-Ernzerhof (PBE) + U + SOC. We obtained Wannier functions using the Wannier90 code [S3], and then calculated the Hall conductivity using WannierBerri code [S4]. A minimal tight-binding model was built based on the hopping parameters derived from the effective Wannier Hamiltonian with the Cr t_{2g} bands in the ferromagnetic state. We have conducted the calculation using the full-potential FPLO code, version fplo18.00-52 (http://www.fplo.de) [S5, S6], within the localized spin density approximation (LSDA) with Perdew-Wang-92 exchange-correlation functional [S7].

II. TEMPERATURE DEPENDENCE OF IN-PLANE RESISTIVITY AND THE ANISOTROPY

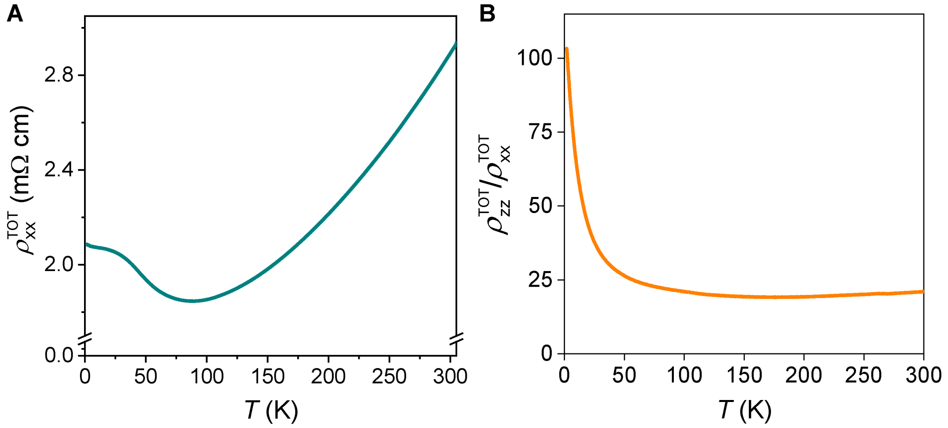

FIG. S2. (*A*) Temperature dependence of resistivity in the *ab*-plane of $AgCrSe_2$. (*B*) Temperature dependence of the resistive anisotropy.

We have measured the T dependence of resistivity along different crystallographic directions, as plotted in Fig. S2. The in-plane resistivity ρ_{xx}^{TOT} displays metallic behavior below room temperature and increases slightly at low temperatures. The resistive anisotropy $\rho_{zz}^{TOT}/\rho_{xx}^{TOT}$ (plotted in Fig. S2B) is large, rising from a factor of 25 at temperatures above 50 K to 100 at 2 K. The large transport anisotropy clearly demonstrates the quasi-two dimensional transport characteristic.

III. OUT-OF-PLANE SPIN CANTING IN AGCRSE$_2$

We have performed magnetization measurements on as-grown single crystals. The magnetization data, with the field aligned to the c-axis direction, are shown in Fig. S3. The M vs H curves exhibit linear relations with a nearly zero net magnetic moment at zero field (Fig. S3A). A careful inspection of the magnetization data around the zero magnetic field actually reveals a weak out-of-plane ferromagnetic moment at low temperatures. The weak magnetic moment could be extracted by subtracting the linear background which is due to magnetic-field-aligned spin-canting. As shown in Fig. S3B by the ΔM vs H curves, a weak magnetization $\sim$ 2.2 mμ_B (i.e. $10^{-3}\mu_B$) per formula unit is detected at 2 K. The magnitude of the magnetization decreases upon T rising, and vanishes at temperatures above 100 K (Fig. S3C). An independent measurement on another crystal (sample B) yielded a maximum of $\sim$ 13 mμ_B per formula unit at 2 K, and the magnetization shows similar temperature dependent behavior (Figs. S3D-F).

Similar weak ferromagnetic features have been reported in other antiferromagnets such as Mn_3Sn [S8], Mn_3Ge [S9] and $CoNb_3S_6$ [S10], and ascribed to a weak spontaneous out-of-plane spin canting. 2.2-13 mμ_B corresponds to a spin canting angle of 0.1° to 0.5° relative to the x-y plane.

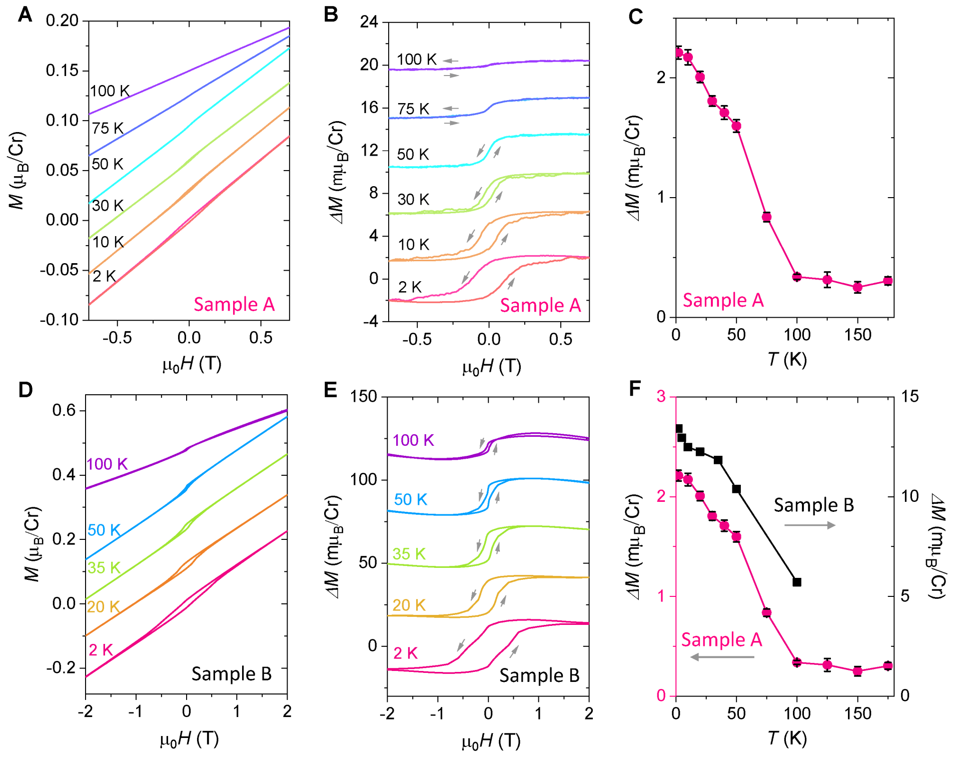

FIG. S3. Out-of-plane spin canting in $AgCrSe_2$. (A) Magnetic field dependence of the magnetization M of sample A at various temperatures, with the field orientated along the c-axis. (B) The out-of plane spin canting can be probed by the uncompensated magnetization ΔM of sample A acquired by removing the linear background. (C) Temperature dependence of the uncompensated magnetization in sample A. (D) M vs H of sample B. (E) ΔM vs H of sample B. (F) Temperature dependence of the uncompensated magnetization of both sample A and B. The curves in (A), (B), (D) and (E) are offset vertically for clarity.

Since the inversion symmetry is broken in the crystal, there can be antisymmetric exchange interactions between the neighboring spins, namely Dzyaloshinsky-Moriya interaction, which favors a spin canting. The out-of-plane spin canting can, in principle, break symmetries in the crystal and give rise to a ferromagnetic behavior. However, as discussed in the main text, an additional component in the Hall resistivity is still observed after taking into the moment into account. Morover, the resulting net moment alone cannot fully explain the observed large hysteresis loop in the Hall resistivity.

IV. NOTE ON THE CONSEQUENCES OF CONDUCTIVITY TENSOR INVERSION

Since $\sigma_{yx}^A = \rho_{xy}^A/((\rho_{xy}^A)^2 + (\rho_{xx}^{TOT})^2)$ (see the main text for definitions), it is tempting to think that if scattering changes ρ_{xx}^{TOT}, it will change the observed σ_{yx}^A. Indeed, such statements have been made in a number of recent publications, in spite of the fact that the σ_{yx}^A under discussion is fundamentally independent of scattering!

The resolution to this apparent paradox is that any calculated σ_{yx}^A based on band Berry curvature would not change, because in reality the change in scattering will affect ρ_{xy}^A.

To see this, note that $\rho_{xy}^A = \sigma_{yx}^A/((\sigma_{xx}^{TOT})^2 + (\sigma_{yx}^A)^2)$ and under the conditions stated in this paper (which apply to other low carrier density AHE systems), $\rho_{xx}^{TOT} \gg \rho_{xy}^A \Rightarrow \sigma_{xx}^{TOT} \gg \sigma_{yx}^A$ and $\rho_{xy}^A = \sigma_{yx}^A/(\sigma_{xx}^{TOT})^2$ and $\sigma_{xx}^{TOT} = (\rho_{xx}^{TOT})^{-1}$. Doubling ρ_{xx}^{TOT} by increasing scattering therefore increases ρ_{xy}^A by a factor of four, leaving σ_{yx}^A unchanged. The consequence of this is that if correct measurements of ρ_{xx}^{TOT} and ρ_{xy}^A are made, and correctly inverted to give σ_{yx}^A, then that value is the intrinsic one for the relevant band and band filling. Any statement that it would somehow be closer to a quantized value if the band filling remained unchanged but the scattering decreased is simply incorrect.

V. THICKNESS DEPENDENCE OF THE AHE

In Fig. 3 of the main text, we have shown the angular dependence of the AHE, with the current applied in the *ab*-plane and the magnetic field rotating relative to the *c*-axis. At first sight, such an observation seems to be consistent with a quantized topological response of some kind, in which ρ_{xy}^A is fixed at a topologically-controlled value, insensitive to changes in external conditions. However, the simplest interpretations of this kind are inconsistent with the size of the AHE that we observe. A topological contribution to the AHE in a two-dimensional system leads to a quantized conductance in units of e^2/h, where e is the electronic charge and h is the Planck constant. Although our measured ρ_{xy}^A is large—comparable with the largest seen in any compensated magnetic system [S8–S11]—inverting the resistivity tensor and converting to two dimensions by factoring out the layer separation of 7 Å gives $\sigma_{yx}^A \cong 7 \times 10^{-4} e^2/h$ per atomic layer. This low value of σ_{yx}^A means that we are far from a topological response based on any bulk physics of $AgCrSe_2$.

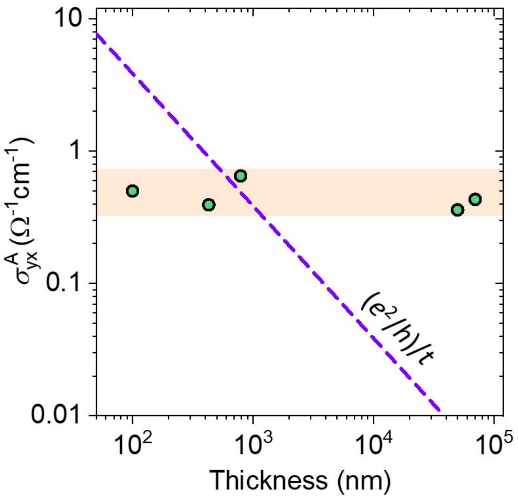

FIG. S4. Thickness dependence of σ_{yx}^A. The dashed line is the quantum conductance e^2/h normalized by the thickness t.

However, one other form of topological response is worthy of consideration. A net non-zero Chern number due to surfaces could result in the reduction of the observed σ_{yx}^A from the quantized value by the ratio n_s/n_b, where n_s is the number of topologically non-trivial layers at or near the sample surface and n_b is the number of topologically trivial bulk layers. This intriguing hypothesis can be checked experimentally by studying samples of varying thickness. We did this, with the results shown in Fig. S4. The observed σ_{yx}^A shows no systematic dependence on sample thickness, and hence none on n_s/n_b. We therefore believe that our observation is that of a non-quantized σ_{yx}^A whose origin is in the bulk physics of $AgCrSe_2$.

VI. ESTIMATION OF R_0 AND R_s

In the main text, we have discussed how ρ_{xy}^0 and ρ_{xy}^A were extracted from ρ_{xy}^{TOT} at 2 K. Here we provide additional data taken at higher temperatures, and estimate the ordinary and anomalous Hall coefficients R_0 and R_s.

As discussed in the main text, the Hall resistivity in $AgCrSe_2$ can be described by $\rho_{xy}^{TOT}(H) = R_0\mu_0 H + \rho_{xy}^A$. The linear relation of $\rho_{xy}^{TOT}(H)$ as a function of H allows us to extract R_0 from the slope, which is obtained to be 7×10^{-8} m^3/C at 2 K, corresponding to a carrier density of 9×10^{19} cm^{-3}. We treat the carrier density to be a constant for temperatures lower than 50 K, and use it to estimate the Fermi level in our electronic structure calculations, employing a rigid band approximation. Electron probe analyses of our crystals show Ag deficit and Cr surplus, in a ratio close to 3:1. Our observation of a relatively low density of hole carriers is consistent with the true ratio being slightly greater than 3:1 in the crystals used for our transport measurements. At each temperature, $R_0\mu_0 H$ was first subtracted from $\rho_{xy}^{TOT}(H)$, and the anomalous Hall resistivity term $\rho_{xy}^{TOT} - \rho_{xy}^0 = \rho_{xy}^{TOT}(H) - R_0\mu_0 H$ could be obtained. R_s could be further extracted from the $\rho_{xy}^{TOT} - \rho_{xy}^0$ versus M plots, as shown in Figs. S5A, C and E. The

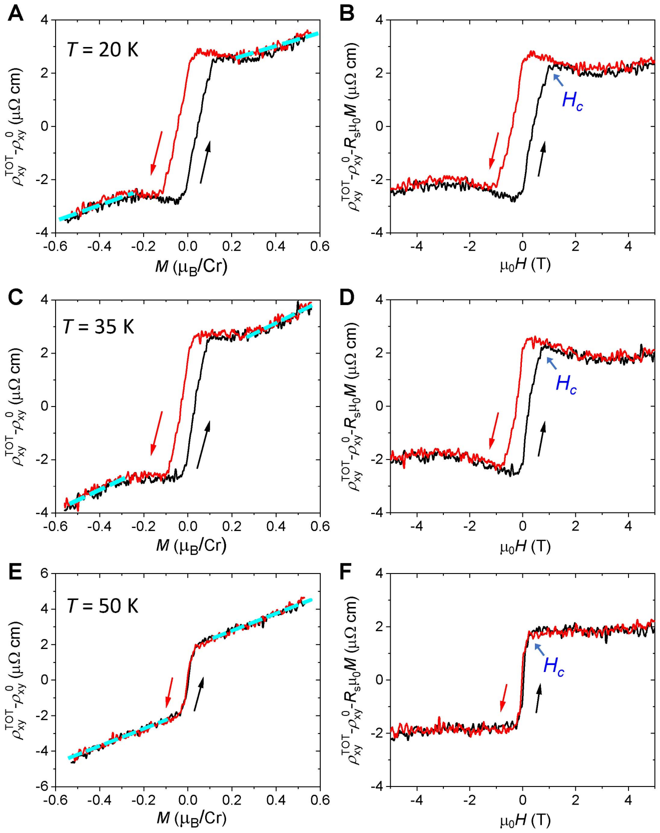

FIG. S5. Additional Hall resistivity data at higher temperatures. (A) (C) and (E) are $\rho_{xy}^{TOT} - \rho_{xy}^{0}$ plotted as a function of M at 20 K, 35 K and 50 K, respectively. (B) (D) and (F) are $\rho_{xy}^{TOT} - \rho_{xy}^{0} - R_s\mu_0 M$ plotted as a function of H at 20 K, 35 K and 50 K, respectively.

dashed lines correspond to the linear contribution from $R_s\mu_0 M$, which originates from the magnetic-field-induced continuous spin-tilting. In the temperature range below 50 K, R_s is found to be $1.5 \sim 3.5 \times 10^{-7}$ m^3/C, which is in fair agreement with those reported in antiferromagnets [S8, S12]. The extra component in ρ_{xy}^{A} could be obtained by removing the contributions from both $R_0\mu_0 H$ and $R_s\mu_0 M$, as plotted in Figs. S5B, D and F. At all temperatures, the extra component is found to be saturating after the resistivity jump, and is nearly independent of H and M.

VII. MAGNETO-TRANSPORT MEASUREMENTS OF A SINGLE-CRYSTAL DEVICE

Besides performing magneto-transport measurements on exfoliated crystals, we also carried out measurements on single crystals, to cross-check the consistency and reproducibility of the transport data.

Fig. S6A shows the anomalous Hall effect data obtained from a single crystal device, for the temperature range below 100 K. The data from the single crystal are fully consistent with those from the exfoliated devices. The Hall resistivity shows a hysteresis loop together with a sizable jump which becomes more pronounced at low T. At 1.8 K, a carrier density of $\sim 3 \times 10^{19}$ cm^{-3} is extracted from the Hall slope near zero magnetic field, and the mobility is estimated to be 25 cm^2/(Vs) employing the Drude model.

We follow the same approach as described in the main text to extract ρ_{xy}^{A} at each temperature (Fig. S6B). At the lowest $T = 1.8$ K, ρ_{xy}^{A} saturates at 3.2±0.1 $\mu\Omega$ cm, which is quantitatively consistent with that obtained from exfoliated crystals.

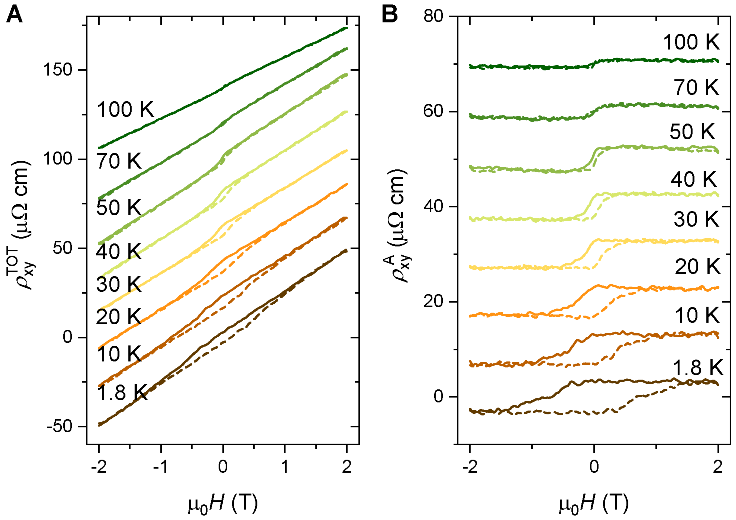

FIG. S6. The Hall resistivity observed in a device realized on a single crystal. (A) Hall resistivity $\rho_{xy}^{TOT}(H)$ taken at temperatures below 100 K. (B) $\rho_{xy}^{A}(H) = \rho_{xy}^{TOT}(H) - R_0\mu_0 H$ extracted from the Hall resistivity. The curves in (A) and (B) are offset vertically for clarity.

VIII. INTERPLAY BETWEEN POLAR STRUCTURE AND MAGNETIC ORDER

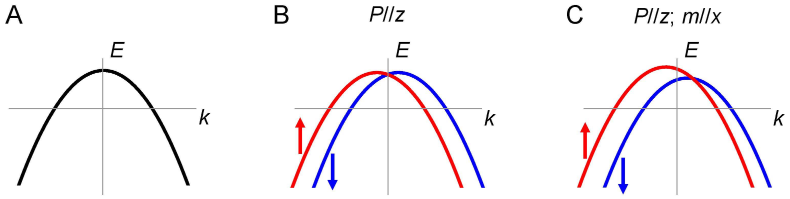

FIG. S7. Schematic of band structures.(A) Band structure with Kramers degeneracy. (B) Rashba-like spin-split band structure. The Kramers degeneracy is lifted as a result of inversion symmetry breaking. (C) Rashba-like spin-split band structure with spins aligned to the x-axis.

Materials with both inversion symmetry and TRS have Kramers spin-degenerate bands (Fig. S7A). Materials with a polar structure lacks the inversion symmetry and this lack of inversion symmetry gives rise to Rashba-like, symmetric spin-splitting in the band structure (Fig. S7B). An in-plane magnetic moment ($m /\!/ x$) introduces asymmetry to the spin-split band structure (Fig. S7C).

IX. THEORETICAL CALCULATIONS OF ANOMALOUS HALL CONDUCTIVITY

To investigate the origin of the AHE in more depth, we have constructed a minimal illustrative tight binding model that describes a monolayer of $AgCrSe_2$ with an approximate cycloidal spin structure and calculated its anomalous Hall conductivity. The key features that such a model needs to incorporate are 1) the noncollinear nature of the cycloidal spin structure in the x-y plane; 2) a parameter to tilt these spins out of the x-y plane; 3) a variable Fermi level and 4) a parameter to introduce a SOC as allowed by the broken inversion symmetry of the real crystal.

To construct such a model while keeping it as simple as possible for illustrative purposes, we consider a triangular-lattice magnetic unit cell (Fig. S8) that consists of three sublattices, whose in-plane-mapped angle of magnetic

moments with respect to the horizontal axis are 0, $-\alpha$ and α respectively. We first calculate the band structure of the ferromagnetic layer using DFT to retain the exchange splitting of the bands, and then approximate it using only three orbitals, d_{xy}, d_{xz} and d_{yz}. To explore that if the AHE is due to the small out-of-plane net magnetization, we have considered two cases: one is without out-of-plane spin canting, i.e. spins are strictly confined to the ab-plane, and the other is with a 0.5° canting to the c-direction.

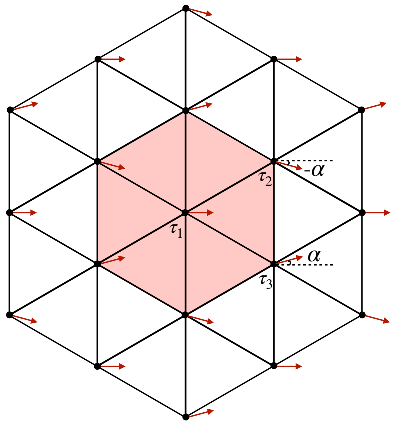

FIG. S8. The magnetic unit cell (pink shaded) with three sublattices.

At zero applied field, the effective Hamiltonian consists of two primary terms: $H = H_0 + H_R$, where H_0 describes the hoppings and H_R is the symmetry allowed SOC term.

The kinetic term is

$$\begin{aligned} H_0 = \sum_{\mathbf{k},s} \Big[\sum_{i=1}^{3} t_0^s c^\dagger_{\mathbf{k},\tau_i,s} c_{\mathbf{k},\tau_i,s} + T^s_{21}(\mathbf{k}) c^\dagger_{\mathbf{k},\tau_2,s} c_{\mathbf{k},\tau_1,s} \\ + T^s_{31}(\mathbf{k}) c^\dagger_{\mathbf{k},\tau_3,s} c_{\mathbf{k},\tau_1,s} + T^s_{32}(\mathbf{k}) c^\dagger_{\mathbf{k},\tau_3,s} c_{\mathbf{k},\tau_2,s} + h.c. \Big], \end{aligned} \tag{S1}$$

$s =\uparrow,\downarrow$ label spins, $\tau_i (i = 1, 2, 3)$ are three sublattices. Inter-sublattice tunnelings are

$$\begin{aligned} T^s(\mathbf{k}) \equiv T^s_{21}(\mathbf{k}) = t^s_{\mathbf{a}_1} e^{-i\mathbf{k}\cdot\mathbf{a}_1} + t^s_{\mathbf{a}_2} e^{i\mathbf{k}\cdot\mathbf{a}_2} + t^s_{\mathbf{a}_3} e^{i\mathbf{k}\cdot\mathbf{a}_3}, \\ T^s_{21}(\mathbf{k}) = T^s_{32}(\mathbf{k}) = [T^s_{31}(\mathbf{k})]^*. \end{aligned} \tag{S2}$$

The hopping parameters in Eq. (S2) are summerized in Table. S1. The hopping lattice vectors are defined as

$$\begin{aligned} \mathbf{a}_1 &= (\frac{\sqrt{3}}{2}, \frac{1}{2})a, \\ \mathbf{a}_2 &= (\frac{\sqrt{3}}{2}, -\frac{1}{2})a, \\ \mathbf{a}_3 &= \mathbf{a}_1 - \mathbf{a}_2. \end{aligned} \tag{S3}$$

where a is the lattice constant of the original triangular lattices.

The kinetic Hamiltonian can be written as

$$H_0(\mathbf{k}) = \sigma_0 \otimes h_0(\mathbf{k}) + \sigma_z \otimes h_z(\mathbf{k}), \tag{S4}$$

where $h_0(\mathbf{k})$ describes hoppings and $h_z(\mathbf{k})$ is the effective exchange field from the assumed out-of-plane Cr ferro-

TABLE S1. Hopping parameters in Eq. (S2).

	$t_0^\uparrow$	$t_{\mathbf{a}_1}^\uparrow$	$t_{\mathbf{a}_2}^\uparrow$	$t_{\mathbf{a}_3}^\uparrow$	$t_0^\downarrow$	$t_{\mathbf{a}_1}^\downarrow$	$t_{\mathbf{a}_2}^\downarrow$	$t_{\mathbf{a}_3}^\downarrow$
d_{xy}	-0.88	0.085	0.085	0	1.291	0.071	0.071	-0.217
d_{xz}	-0.88	0.085	0	0.085	1.291	0.071	-0.217	0.071
d_{yz}	-0.88	0	0.085	0.085	1.291	-0.217	0.071	0.071

magnetic order. h_0 and h_z are 3×3 matrices

$$\begin{aligned} h_0(\mathbf{k}) &= \frac{1}{2}(h^\uparrow + h^\downarrow), \\ h_z(\mathbf{k}) &= \frac{1}{2}(h^\uparrow - h^\downarrow), \\ h^s(\mathbf{k}) &= \begin{pmatrix} t_0^s & T^{s*}(\mathbf{k}) & T^s(\mathbf{k}) \\ T^s(\mathbf{k}) & t_0^s & T^{s*}(\mathbf{k}) \\ T^{s*}(\mathbf{k}) & T^s(\mathbf{k}) & t_0^s \end{pmatrix}. \end{aligned} \tag{S5}$$

In the actual crystal, Cr moments are largely aligned in-plane in the ground state and a small canted moment on the order of milli-μ_B was observed experimentally. To model the noncollinear magnetic order shown in Fig. S7 and the symmetry allowed small spin canting, we rewrite the Hamiltonian $H_0(\mathbf{k})$ to be

$$\begin{aligned} H_0(\mathbf{k}) = \sigma_0 \otimes h_0(\mathbf{k}) + \sigma_z \otimes \beta h_z^d(\mathbf{k}) + \sigma_x \otimes \bar{\beta} h_z^d(\mathbf{k}) \begin{pmatrix} 1 & 0 & 0 \\ 0 & \cos\alpha & 0 \\ 0 & 0 & \cos\alpha \end{pmatrix} \\ + \sigma_y \otimes \bar{\beta} h_z^d(\mathbf{k}) \begin{pmatrix} 0 & 0 & 0 \\ 0 & -\sin\alpha & 0 \\ 0 & 0 & \sin\alpha \end{pmatrix} \end{aligned} \tag{S6}$$

The phenomenological parameter β models the spin tilting at angle θ with respect to the crystal c-axis. β and $\bar{\beta}$ satisfy $\beta(\theta = \pi/2) = 0$ and $\bar{\beta} = \sqrt{1-\beta^2}$. $h_z^d(\mathbf{k})$ only keeps the diagonal part of $h_z(\mathbf{k})$, i.e. ignoring the SOC-like term when we enlarge the magnetic unit cell. Therefore $h_z^d(\mathbf{k}) = (t_0^\uparrow - t_0^\downarrow)/2 \; I_{3\times3}$. The terms depend on angle α are just the sublattice-dependent on-site exchange field along x- and y-directions. Specifically,

$$\begin{aligned} H_0(\mathbf{k}) = \sigma_0 \otimes h_0(\mathbf{k}) + \sigma_z \otimes \frac{\beta}{2}(t_0^\uparrow - t_0^\downarrow) I_{3\times3} + \sigma_x \otimes \frac{\bar{\beta}}{2}(t_0^\uparrow - t_0^\downarrow) \begin{pmatrix} 1 & 0 & 0 \\ 0 & \cos\alpha & 0 \\ 0 & 0 & \cos\alpha \end{pmatrix} \\ + \sigma_y \otimes \frac{\bar{\beta}}{2}(t_0^\uparrow - t_0^\downarrow) \begin{pmatrix} 0 & 0 & 0 \\ 0 & -\sin\alpha & 0 \\ 0 & 0 & \sin\alpha \end{pmatrix} \end{aligned} \tag{S7}$$

The Rashba SOC term of a triangular lattice can be derived from the general tight-binding formula

$$H_R = it_R \sum_{\langle i\gamma, j\lambda\rangle ss'} \hat{z} \cdot (\mathbf{d}_{i\gamma,j\lambda} \times \boldsymbol{\sigma}_{ss'}) c_{i\gamma s}^\dagger c_{j\lambda s'}, \tag{S8}$$

where i, j label unit cells, γ, λ label sublattices and s, s' label spins. The vector $\mathbf{d}_{i\alpha,j\beta} = \mathbf{R}_{j\beta} - \mathbf{R}_{i\alpha}$ points from lattice site $\mathbf{R}_{i\alpha}$ to $\mathbf{R}_{j\beta}$. Transform to momentum space,

$$\begin{aligned} H_R = it_R \sum_{\mathbf{k},s,s'} \Big[\hat{z} \cdot \mathbf{h}_R(\mathbf{k}) \times \boldsymbol{\sigma}_{ss'} \big(c_{\mathbf{k},\tau_1,s}^\dagger c_{\mathbf{k},\tau_2,s'} + c_{\mathbf{k},\tau_2,s}^\dagger c_{\mathbf{k},\tau_3,s'} + c_{\mathbf{k},\tau_3,s}^\dagger c_{\mathbf{k},\tau_1,s'} \big) \\ - \hat{z} \cdot \mathbf{h}_R^*(\mathbf{k}) \times \boldsymbol{\sigma}_{ss'} \big(c_{\mathbf{k},\tau_2,s}^\dagger c_{\mathbf{k},\tau_1,s'} + c_{\mathbf{k},\tau_3,s}^\dagger c_{\mathbf{k},\tau_2,s'} + c_{\mathbf{k},\tau_1,s}^\dagger c_{\mathbf{k},\tau_3,s'} \big) \Big], \end{aligned} \tag{S9}$$

where the complex in-plane vector

$$H_R(\mathbf{k}) = e^{i\mathbf{k}\cdot\mathbf{a}_1}\mathbf{a}_1 - e^{-i\mathbf{k}\cdot\mathbf{a}_2}\mathbf{a}_2 - e^{-i\mathbf{k}\cdot\mathbf{a}_3}\mathbf{a}_3. \tag{S10}$$

Therefore,

$$H_R(\mathbf{k}) = it_R\left[\sigma_x \otimes \begin{pmatrix} 0 & -h_{R,y} & h^*_{R,y} \\ h^*_{R,y} & 0 & -h_{R,y} \\ -h_{R,y} & h^*_{R,y} & 0 \end{pmatrix} + \sigma_y \otimes \begin{pmatrix} 0 & h_{R,x} & -h^*_{R,x} \\ -h^*_{R,x} & 0 & h_{R,x} \\ h_{R,x} & -h^*_{R,x} & 0 \end{pmatrix}\right] \tag{S11}$$

Because H_0 in Eq. (S7) includes both kinetic and exchange field terms, the full Hamiltonian is the sum of Eq. (S7) and Eq. (S11)

$$H(\mathbf{k}) = H_0(\mathbf{k}) + H_R(\mathbf{k}). \tag{S12}$$

The Berry curvature Ω^m_{xy} of the mth band in our model was calculated using the standard Kubo formula:

$$\Omega^m_{xy}(\boldsymbol{k}) = -\mathrm{Im}\sum_{m\neq n}\frac{\langle m|\nabla_{k_x}H|n\rangle\langle n|\nabla_{k_y}H|m\rangle - (m \leftrightarrow n)}{(E_m - E_n)^2}\ , \tag{S13}$$

where $|m\rangle$ and E_m ($|n\rangle$ and E_n) are the Bloch eigenstate and eigenvalue respectively, for the mth (nth) band at momentum $\boldsymbol{k}$.

The anomalous Hall conductivity σ^A_{yx} in our model was calculated as

$$\sigma^A_{yx} = -\frac{e^2}{\hbar}\int\frac{d^2\boldsymbol{k}}{(2\pi)^2}\,\Omega_{xy}(\boldsymbol{k})\ , \tag{S14}$$

where e is the electronic charge, and $\Omega_{xy}(\boldsymbol{k}) = \sum_m \Omega^m_{xy}(\boldsymbol{k})$ is the sum of Berry curvatures over the occupied bands.

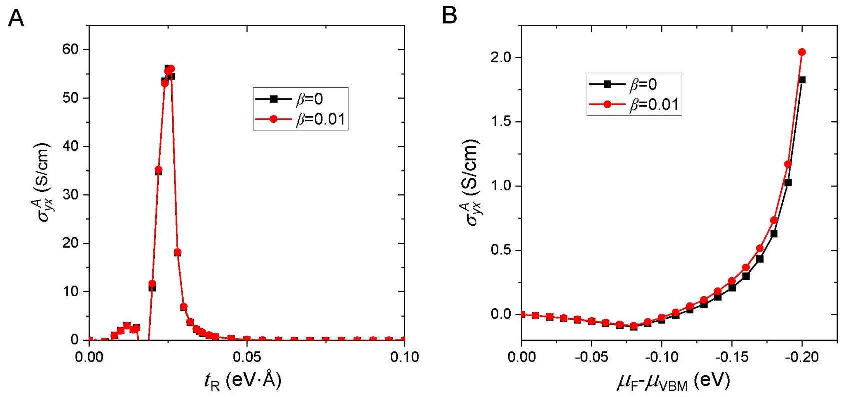

FIG. S9. Anomalous Hall conductivity for a monolayer with noncollinear spin structure computed using the full tight-binding model described in the text, with $\alpha = 15°$. (A) Anomalous Hall conductivity as a function of t_R with $\beta = 0$ and $\beta = 0.01$ respectively, at $\mu_F - \mu_{\mathrm{VBM}} = -0.13$ eV. (B) Anomalous Hall conductivity as a function of $\mu_F - \mu_{\mathrm{VBM}}$ with $t_R = 0.05$ eV $\cdot$ Å, and $\beta = 0$ and $\beta = 0.01$ respectively. Note that we observed the canted angle of Cr moments is around $0.2° - 0.5°$, so we set $\beta = 0.01 \sim \sin(0.5°)$ in the calculations to be consistent with the experiment.

The anomalous Hall conductivity was calculated using the full Hamiltonian by varying the Rashba SOC strength t_R and the Fermi level μ_F with respect to the valence band maximum (VBM) μ_{VBM}, and the results are shown in Fig. S9. Spin structures without out-of-plane spin tilting ($\beta = 0$) and with a $0.5°$ out-of-plane spin tilting ($\beta = 0.1$)

are both considered. Using a Fermi level estimated from the carrier concentration, calculations reveal that a non-zero Berry curvature occurs when t_R is non-zero, even when the spins are strictly confined to be in the ab-plane ($\beta = 0$). As shown in Fig. S9, by taking $\alpha = 15°$ which is close to the rotation angle between the adjacent spins in the actual crystal, the model yields both the correct order of magnitude of anomalous Hall conductivity ($\cong$ 1 S/cm) and captures its evolution with a changing μ_F across the range probed by the gating experiments. The out-of-plane spin tilting can lead to a higher anomalous Hall conductivity, but only change the magnitude slightly.

[S1] M. Baenitz, M. M. Piva, S. Luther, J. Sichelschmidt, K. M. Ranjith, H. Dawczak-Debicki, M. O. Ajeesh, S.-J. Kim, G. Siemann, C. Bigi, P. Manuel, D. Khalyavin, D. A. Sokolov, P. Mokhtari, H. Zhang, H. Yasuoka, P. D. C. King, G. Vinai, V. Polewczyk, P. Torelli, J. Wosnitza, U. Burkhardt, B. Schmidt, H. Rosner, S. Wirth, H. Kühne, M. Nicklas, and M. Schmidt, Planar triangular $S = 3/2$ magnet $AgCrSe_2$: Magnetic frustration, short range correlations, and field-tuned anisotropic cycloidal magnetic order, Phys. Rev. B **104**, 134410 (2021).

[S2] G. Kresse and J. Furthmüller, Efficient iterative schemes for ab initio total-energy calculations using a plane-wave basis set, Phys. Rev. B **54**, 11169 (1996).

[S3] A. A. Mostofi, J. R. Yates, G. Pizzi, Y.-S. Lee, I. Souza, D. Vanderbilt, and N. Marzari, An updated version of wannier90: A tool for obtaining maximally-localised wannier functions, Computer Physics Communications **185**, 2309 (2014).

[S4] S. S. Tsirkin, High performance Wannier interpolation of Berry curvature and related quantities with WannierBerri code, npj Comput Mater **7**, 33 (2021).

[S5] K. Koepernik and H. Eschrig, Full-potential nonorthogonal local-orbital minimum-basis band-structure scheme, Phys. Rev. B **59**, 1743 (1999).

[S6] I. Opahle, K. Koepernik, and H. Eschrig, Full-potential band-structure calculation of iron pyrite, Phys. Rev. B **60**, 14035 (1999).

[S7] J. P. Perdew and Y. Wang, Accurate and simple analytic representation of the electron-gas correlation energy, Phys. Rev. B **45**, 13244 (1992).

[S8] S. Nakatsuji, N. Kiyohara, and T. Higo, Large anomalous Hall effect in a non-collinear antiferromagnet at room temperature, Nature **527**, 212 (2015).

[S9] A. K. Nayak, J. E. Fischer, Y. Sun, B. Yan, J. Karel, A. C. Komarek, C. Shekhar, N. Kumar, W. Schnelle, J. Kübler, *et al.*, Large anomalous Hall effect driven by a nonvanishing Berry curvature in the noncolinear antiferromagnet Mn_3Ge, Sci. Adv. **2**, e1501870 (2016).

[S10] N. J. Ghimire, A. Botana, J. Jiang, J. Zhang, Y.-S. Chen, and J. Mitchell, Large anomalous Hall effect in the chiral-lattice antiferromagnet $CoNb_3S_6$, Nat. Commun. **9**, 1 (2018).

[S11] S. H. Lee, Y. Zhu, Y. Wang, L. Miao, T. Pillsbury, H. Yi, S. Kempinger, J. Hu, C. A. Heikes, P. Quarterman, *et al.*, Spin scattering and noncollinear spin structure-induced intrinsic anomalous Hall effect in antiferromagnetic topological insulator $MnBi_2Te_4$, Phys. Rev. Res. **1**, 012011 (2019).

[S12] H. Takatsu, S. Yonezawa, S. Fujimoto, and Y. Maeno, Unconventional anomalous Hall effect in the metallic triangular-lattice magnet $PdCrO_2$, Phys. Rev. Lett. **105**, 137201 (2010).
